# Regional hypothermia attenuates secondary-injury caused by time-out application of tourniquets following limb fragments injury combined with hemorrhagic shock

**DOI:** 10.1186/s13049-019-0678-3

**Published:** 2019-11-21

**Authors:** Changmei Weng, Kai Lan, Tao Li, Liangchao Zhang, Jianmin Wang, Xinan Lai

**Affiliations:** 1 Research Institute of Surgery, Daping Hospital, Third Military Medical University, Chongqing, 400042 China; 2 State Key Laboratory of Trauma, Burn and Combined Injury Research Institute, Third Military Medical University, Chongqing, 400042 China; 3 State Key Laboratory of Trauma and Burns, Surgery Research Institute; Research Institute of Surgery, Daping Hospital, Third Military Medical University, 10 Changjiang Road, Chongqing, 400042 China; 4 Joint Surgery Center, Southwest Hospital, Third Military Medical University, Chongqing, 400038 China

**Keywords:** Fragment injury, Hemorrhagic shock, Tourniquet, Regional hypothermia, Distant lung injury

## Abstract

**Background:**

Tourniquet is the most widely used and effective first-aid equipment for controlling hemorrhage of injured limb in battlefield. However, time-out application of tourniquets leads to ischemic-necrosis of skeletal muscles and ischemia-reperfusion injury. Regional hypothermia (RH) on wounded limb can relieve the injury on local tissue and distant organs. We aimed to investigate the protective effects of RH on rabbits’ limbs injured by a steel-ball combined with hemorrhagic-shock, and then employed tourniquet over-time, tried to identify the optimal treatment RH.

**Methods:**

Thirty rabbits were randomly divided into 5 groups. All rabbits were anesthetized, intubated femoral artery and vein in right-hind limbs. Sham operation group (**Sham**): only femoral arteriovenous cannula in right-hind limb. None RH group (**NRH**): rabbits were intubated as Sham group, then the soft tissues of rabbits’ left-hinds were injured by a steel-ball shooting, and were exsanguinated until shock, then bundled with rubber tourniquets for 4 h. **Three RH subgroups**: rabbits were injured as mentioned above, the injured limbs were bundled with rubber tourniquets and treated with different temperature (5 ± 1 °C, 10 ± 1 °C, and 20 ± 1 °C, respectively) for 4 h. The injury severity of lung and regional muscle was assessed by histologic examination. Activity of adenosine triphosphatase (ATPase) and content of malondialdehyde (MDA) in muscle, inflammatory cytokines, myoglobin, creatine kinase-MM (CK-MM), Heme, Heme oxygenase 1 (HO-1), lactic acid (Lac), and lectrolyte ion in serum were detected.

**Results:**

Following with RH treatment, the injury of lung and local muscle tissue was alleviated evidencing by mitigation of histopathological changes, significant decrease of water-content and MDA content, and increase of ATPase activity. Lower level of Lac, Potassium (K^+^), inflammatory cytokines, Heme, CK-MM, myoglobin content, and higher level of Calcium (Ca^2+^), HO-1 content were shown in RH treatment. 10 °C was the most effective RH to increase ATPase activity, and decrease MDA, myoglobin, CK-MM content.

**Conclusion:**

Transient RH (4 h) had a “long-term mitigation effects” (continued for 6 h) on time-out application of tourniquet with the fluid resuscitation and core temperature maintenance, and the most effective temperature for reducing the side effects on tourniquet time-out application was 10 °C.

## Background

The incidence of limb injuries has increased greatly in modern war, due to the extensive use of explosive weapon. Limb injuries are also common in terrorist attacks, accidents, and other civilian trauma in daily life. According to the reports, the incidence of injuries to the limbs is much higher (up to 60–70%) than to the other parts of the body, and the percentage of casualties killed in action as a result of exsanguinating hemorrhage of limbs is about 10% [1–3]. The most commonly and also the most effective pre-hospital cared equipment for limb hemostasis was tourniquet [4]. However, the tourniquet application is limited by application-duration and application-occasion. The best time for tourniquet application is before the injured person undergoing hemorrhagic shock, and the safety-time-limit of employing tourniquet is no more than 2 h [5]. The survival analysis of America Army Iraq battlefield demonstrated that using tourniquet to the wounded who had already hemorrhagic shock, the survival rate was only 4% of which using tourniquet before hemorrhagic shock [6]. Side effects such as irreversible injury in muscles and nerves, faster progresses in tissue necrosis are presented in the wounded person who is applied tourniquet out of the safety-limit-time [7–9]. As well as skeletal muscle degeneration, rhabdomyolysis, and release of myoglobin and other toxic substances caused by time-out application of tourniquets on limb which injured by fragments and combined with hemorrhagic shock are important causes of injury to organs such as kidney, lung, and liver [10, 11]. Moreover, once removing tourniquet, damage cascade effects emerge, such as injury of limb and complications of distant organ were aggravated due to reperfusion of limb. Because of the high rate of hemorrhagic shock and traumatic shock in war-wounds or civil injuries, accompanied with bad environment (such as backcountry or battlefield) and limited medical supports, it is very difficult to escort the wounded person to the effective treatment location in limited time. Therefore, it is urgent to improve the application-time of tourniquet.

Previous reports indicated that hypothermia could decrease oxygen consumption and reduce tissue metabolism, and has been applied in many clinical surgeries [12, 13] (such as cardiac arrest [14], brain Injury [15], and acute lung injury [10]). Hypothermia has also been proven to attenuate complications caused by ischemia reperfusion [16, 17]. It is demonstrated that regional hypothermia (RH) could significantly improve the protective effects on both local tissue and distant organ in injured tissues [18, 19]. Interestingly, effective alleviation on distant organs (hepatic, renal and lung) injury was detected when treated with RH (the temperature of regional traumatic muscle was 15–20 °C) on blast injured limb [10, 11]. Therefore, we speculated that appropriate RH also could be employed in injured limbs, and mitigate complications which were caused by time-out application of tourniquet.

As rabbit has moderate physique, and the hind limb skeletal muscle tissue of rabbit is abundant, many studies used the hind limbs of rabbits as the object of study on the safety time limit of tourniquets [20, 21]. Moreover, a number of literatures have also used rabbits as experimental animals to study the characteristics and mechanisms of hemorrhagic shock and ischemia-reperfusion [22–24]. In this study, limb fragment-penetrating trauma for time-out application of tourniquet on rabbit limb was created, with fragment injury combined hemorrhagic shock characteristics, which could simulate of the injured limbs on the battlefield or other civilian trauma accident, and the effect of regional hypothermia in relieving lung injury when using a limb tourniquet over-time were studied. Besides, different temperatures were employed to investigate the most effective RH.

## Methods

### Animal experiments

**Fig. 1:**
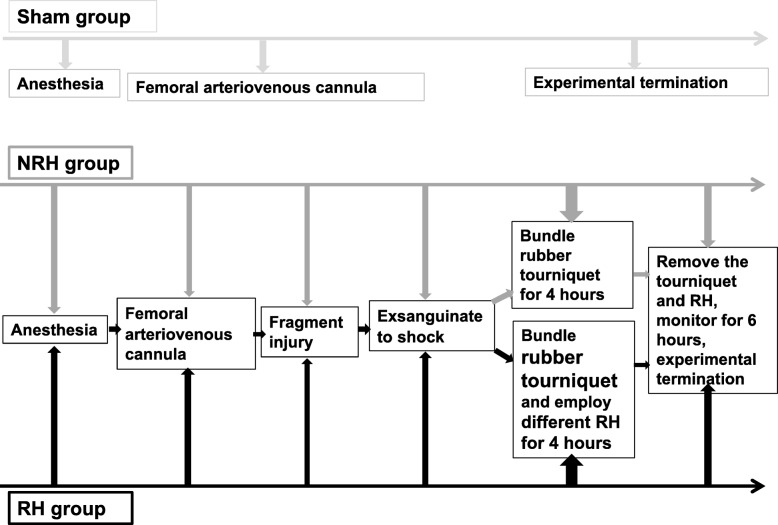
Experimental protocols. All rabbits were anesthetized, intubated femoral artery and vein in right-hind limbs. The soft tissues of rabbits’ left-hinds were injured by a steel-ball shooting, and were exsanguinated until shock, then bundled with rubber tourniquets and treated with different temperature (5 ± 1 °C, 10 ± 1 °C and 20 ± 1 °C, respectively) for 4 h. Then animals were monitored for 6 h until the end of the experiment

The animal experiments were approved by the Ethic Committee of Third Military Medical University and performed in accordance with guidelines of Ethics Committee for Animal Experiments. New Zealand white rabbits (2.0–2.5 kg) of both sexes were housed for 12 h under light-dark conditions with free access to water and standard laboratory chow. After 12 h fasting prior to trauma, all the experimental rabbits were anesthetized with 3% pentobarbital (30 mg/kg of body weight), and divided into 5 groups randomly (*n* = 6 per group), and femoral arteriovenous cannula in right hind limbs were performed for infusion and blood collection. Then the rabbits’ left-hind limbs were shot with a 0.25 g steel-ball from a 7.62 mm smooth-bore rifle (the impacting velocity of the steel bull was about 550 m/s) at middle and lower sections of thighs where muscle is rich, avoiding injury to the femoral arteries and femur. The injured rabbits were exsanguinated to shock and the mean artery pressure (MAP) was 45 ± 5 mmHg. Then the injured limbs were bundled with rubber tourniquets for 4 h (distal arterial artery fluctuations disappearance on injured limbs were detected by ultrasound.). After removal of the tourniquets, the animals were monitored for 6 h until the end of the experiment. Blood transfusion and fluid (Sodium Lactate Ringer’s injection) infusion therapy were taken to all experimental rabbits 1 h after injury for rising MAP to 70 ± 5 mmHg, and the core temperature of rabbits were maintained at normal level (normal rabbit body temperature was 38.0–39.0 °C), which were rewarmed by infrared radiation heating. At the end of experiment, all the rabbits were euthanized by general anesthesia. The experiment procedure was shown in Fig. 1.

**Fig. 2:**
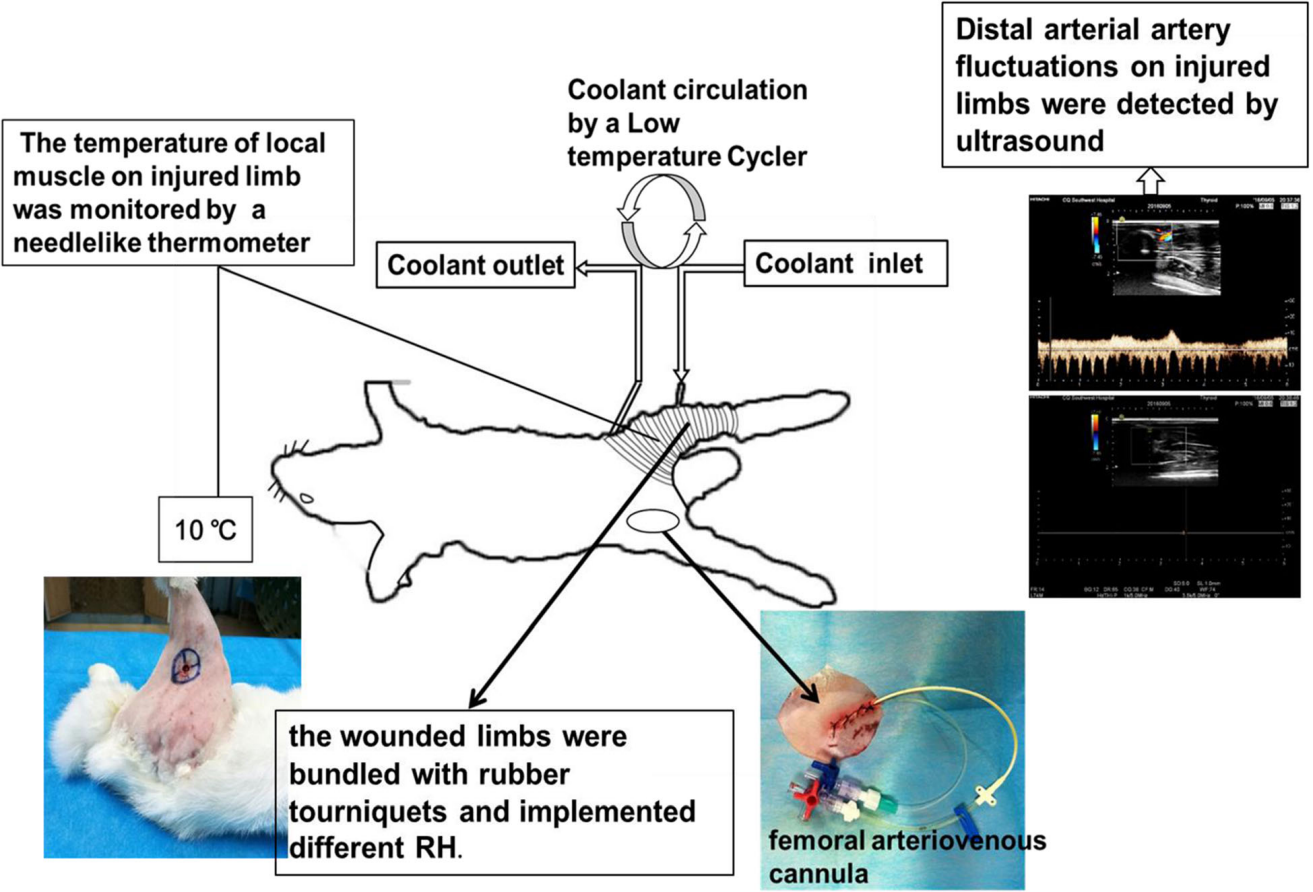
Experiment schematic diagram

Sham operation group (**Sham group**): only femoral arteriovenous cannula in right hind limb for blank control. None regional hypothermia group (**NRH group**): the injured limbs were bundled with rubber tourniquets for 4 h. After removal of the tourniquets, the animals were monitored for 6 h until the end of the experiment. **Three RH subgroups**: the wounded limbs were bundled with rubber tourniquets and at the same time implemented RH for 4 h, the RH of muscle on injured limbs were maintained at 5 ± 1 °C, 10 ± 1 °C and 20 ± 1 °C respectively by a Low Temperature Cycler (HX-08, Jiangsu Tianling Instrument. Jiangsu, China), from which different temperature of coolant was circulated through a rubber conduit, and then reduced the local temperature of the injured muscle. A needlelike thermometer was inserted into the muscle tissue to monitor temperature (Fig. 2). After removal of the tourniquets and RH, the animals were monitored for 6 h until the end of the experiment.

### Related vital signs monitoring and arterial blood gas analysis

Arterial blood pressure, anus temperature, breathing, and heart rate were monitored with Power Lab Monitor (PHILIPS sure signs VM8, Amsterdam, Netherlands) throughout the experiments. Arterial blood was sampled for blood gas analysis via a portable blood gas analyzer (Abbott Company. Chicago, USA) at the time point: before injury (T_0_), and 2-h (T_1_), 4-h (T_2_), 6-h (T_3_) after removing the tourniquet.

### Blood and tissue sampling detection

Arterial blood was sampled at the time point mentioned above for collecting serum and detecting inflammatory cytokines (TNF-α, IL-2, IL-6), myoglobin, creatine kinase-MM (CK-MM), Heme, and Heme oxygenase 1 (HO-1). The specific method referred to the manual of relevant Elisa kits (Xitang Biological Technology, Shanghai, China).

At the end of experiment, all the rabbits were euthanized by general anesthesia. Skeletal muscle of injured limb and lower lobe of right lung were sampled and divided into three parts: one was used for determining tissue water content, one was fixed with 4% paraformaldehyde for executing histologic examination, and one was stored in liquid nitrogen for western blot or Elisa determination.

### Lung and muscle water content determination

The samples of lung and muscle were completely dehydrated for 72 h in an oven at 60 °C. The lung and muscle weight was measured before and after dehydration, and the water content was calculated with the formula (wet /dry weight).

### Histologic examination and immunohistochemistry

The samples of lung and muscle were fixed in 4% paraformaldehyde for at least 48 h, then embedded in paraffin wax, sectioned into slices with 5-μm thickness, stained with hematoxylin and eosin, and finally examined with microscope. The traumatic lung histopathological changes including infiltration of the inflammatory cells, lung edema, hemorrhage and pulmonary architecture were evaluated. The severity of acute lung injury (ALI) was scored according to categorical degree scoring injury from 0 (minimal or no damage) to 4 (severe damage) as described previously [25, 26], and the mean score of 5 random areas in each section per animal was used for data analysis.

Immunohistochemistry staining for detection of HO-1 was performed as the instruction manual (Maixin Biologicals, Fuzhou, China). In brief, the slides were cleared of paraffin and subjected to antigen retrieval, then quenched of endogenous peroxidase activity with endogenous peroxidase blocker, the primary antibody rabbit anti- HO-1 (Novus Biologicals, Colorado, USA) was employed under 4 °C for overnight. The secondary antibody (Abcam, Cambridge, UK) was recognized using a horseradish peroxidase-conjugated goat anti-rabbit/IgG and a DAB kit (Beyotime Biotechnology, Shanghai, China). The images were observed using Olympus microscope BX50.

### Western blot and tissue Elisa

Both lungs and muscles were stored in liquid nitrogen. The muscles were grinded with liquid nitrogen to collect tissue homogenate for detecting the concentration of malondialdehyde (MDA) and adenosine triphosphatase (ATPase) activity via Elisa, and the specific method referred to the manual of relevant Elisa kits (Nanjing Jiancheng Bioengineering Institute, Nanjing, China).

The samples of lung were also grinded with liquid nitrogen to extract protein for detecting HO-1 via western bolt. The total protein for western blot was obtained following the manufacturer’s instructions of the total protein extraction kit (Key GEN Bio TECH. Jiangsu, China). Equal amounts of protein samples (about 50 μg) were separated on 10% sodium do decyl sulfate (SDS)-polyacrylamide gels and electrophoretic ally transferred to PVDF membranes. Following blocking with 5% non-fat milk at room temperature for 2 h, membranes were incubated with the primary antibody dilution of (HO-1 1:1000, Tubulin 1:3500) overnight at 4 °C and then incubated with a horseradish peroxidase-conjugated secondary antibody (1:10000) for 2 h at room temperature. Specific immune complexes were detected using ImmobionTM Western Chemiluminescent HRP Substrate (Millipore, MA, USA). The band’s density was also quantified using Image-J (National Institutes of Health, MD, USA). The optical density of each protein was normalized to tubulin.

### Statistical analysis

The data were presented as mean ± SD (standard deviation), unless indicated otherwise, and the data were analyzed using analysis of variance followed by SPSS software version 23 (IBM Corporation, NY, USA) and Tukey comparison (Graph pad software Inc., La Jolla, CA). *p* < 0.05 was considered to be statistical significant.

## Results

In this study, limb fragment injury combined with hemorrhagic shock and employed tourniquet was generated, which simulated the injured limbs on battlefield or clinical emergency accident. The anal temperatures of all experimental rabbits were kept at normal ranges throughout the experiment (Data were shown in Additional file 1: Table S1) to avoid trauma “triad of death”, which describes the combination of acidosis, coagulopathy, and hypothermia, can cause death of the patient. The results indicated that with the fluid resuscitation and core temperature maintenance, the experimental rabbits could survive for more than 10 h after subjecting to hemorrhagic shock on limbs and then treating with tourniquet for 4 h.

### The effects of RH on lung and local muscle histopathology

**Fig. 3:**
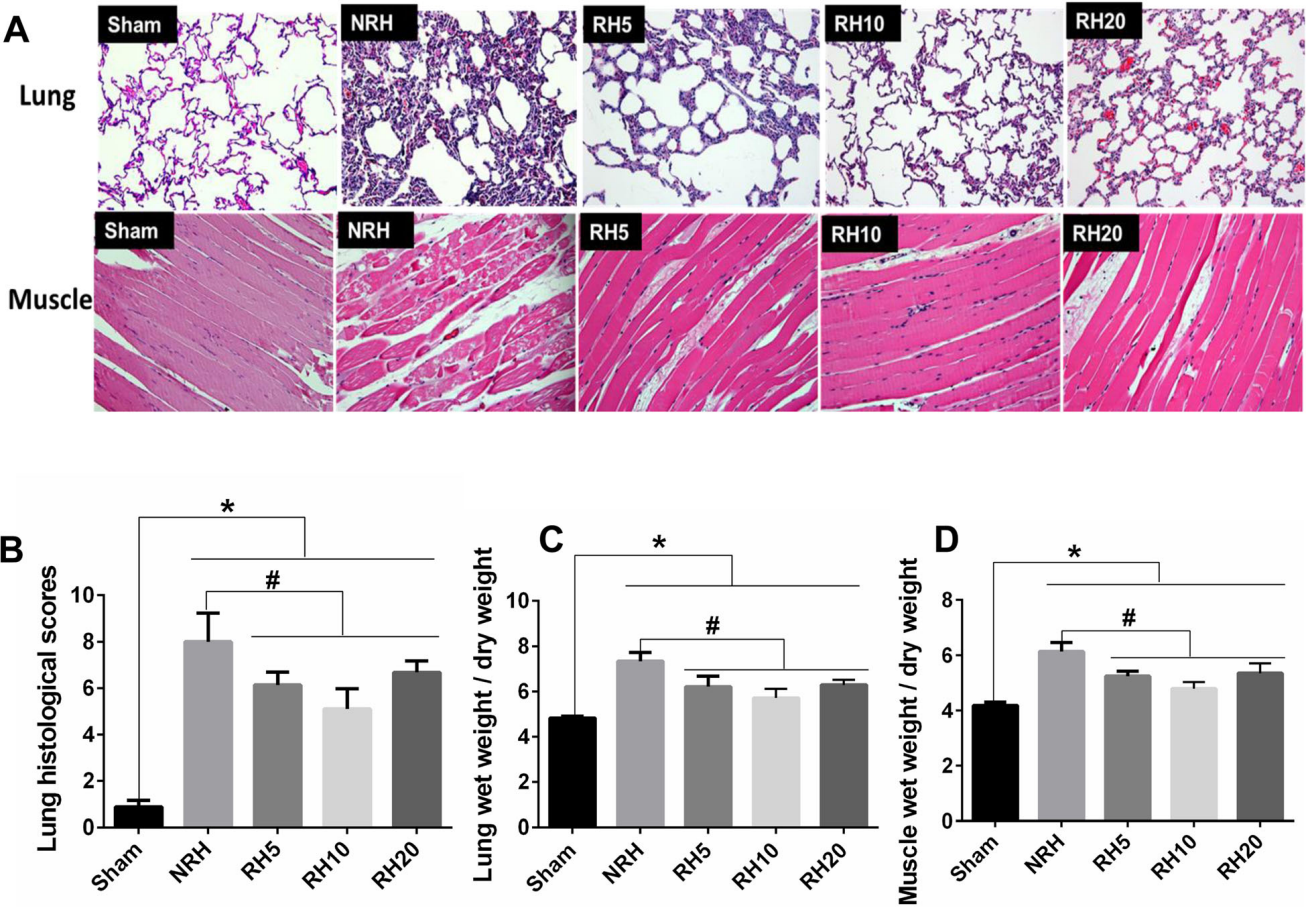
The effects of RH on lung and regional muscle histopathology. **a**. Histopathology changes of the lung and regional injured muscle following different RH treatment. Representative microphotographs (H&E, 20×) were taken from the Sham, NRH, RH5°C, RH10°C and RH20°C, respectively. **b**. Histopathological scoring data of lung injury. **c**. Lung water content was represented by wet weight/dry weight. **d**. Muscle water content was represented by wet weight/dry weight. **Notes:** Data are mean ± SD (*n* = 6). Sham: Sham group. NRH: None Regional hypothermia. RH5: Regional hypothermia 5 °C. RH10: Regional hypothermia 10 °C. RH20: Regional hypothermia 20 °C. *p < 0.05 vs. Sham, #*p* < 0.05 vs. NRH

The histological structure of lung and injured muscle was shown in Fig. 3a. In contrast to the blank control group (Sham group), different degrees of alveolar structural damage were detected both in NRH and RH groups. Lung histological changes including congestion, hemorrhage, alveolar wall thickening, and inflammatory cells were significantly attenuated in RH groups compared with that in NRH group, and scores of the Smith Semi Micro Quantitative Analysis were decreased in RH groups compared with NRH group (#*p* < 0.05) (Fig. 3b). Local muscle was edema and deformation, muscle gap was obviously widened, and the structure of myofibril was unclear in NRH group. Different degrees of edema were found in muscle fiber among the three RH groups, while the RH10 group was the lightest.

Lung wet/dry weight ratio significantly increased in the NRH and RH groups compared with that of sham group. Moreover, the values of three RH groups all decreased compared with that of NRH group (#*p* < 0.05) (Fig. 3c). Similar results were detected in muscle wet/dry weight ratio (#*p* < 0.05) (Fig. 3d). All these results demonstrated that RH could attenuate the lung and local muscle injures of rabbits, whose limbs were experienced a steel-ball penetrating injury combined with hemorrhagic shock and applied with tourniquet, the effects of RH10 group were most prominent.

### Physiologic variables

In order to simulate fragment injury combined with hemorrhagic shock, each of the experimental rabbits except sham group were shot by a steel-ball, and then exsanguinated to shock (MAP was 45 ± 5 mmHg). Blood transfusion and fluid infusion therapy were implemented 1 h after injury. Anal temperature and heart rate of all rabbits were kept at normal ranges throughout the experiments. After removing tourniquet, the breath rate of NRH group increased significantly (#*p* < 0.05) compared with Sham and RH groups, however the MAP in NRH group was significantly lower (#*p* < 0.05) than Sham and RH groups (Data were shown in Additional file 1: Table S1).

### Blood gas analysis of arterial blood

Arterial blood sample of rabbits in all groups were collected at the time before injury (T_0_), and after removing the tourniquet (T_1_, T_2_, T_3_) to analyze blood gas. The pH, SaO_2_, HCO_3_^−^, and PaCO_2_ of all rabbits were kept at normal ranges throughout the experiment (Data were shown in Additional file 1: Table S1).

**Fig. 4:**
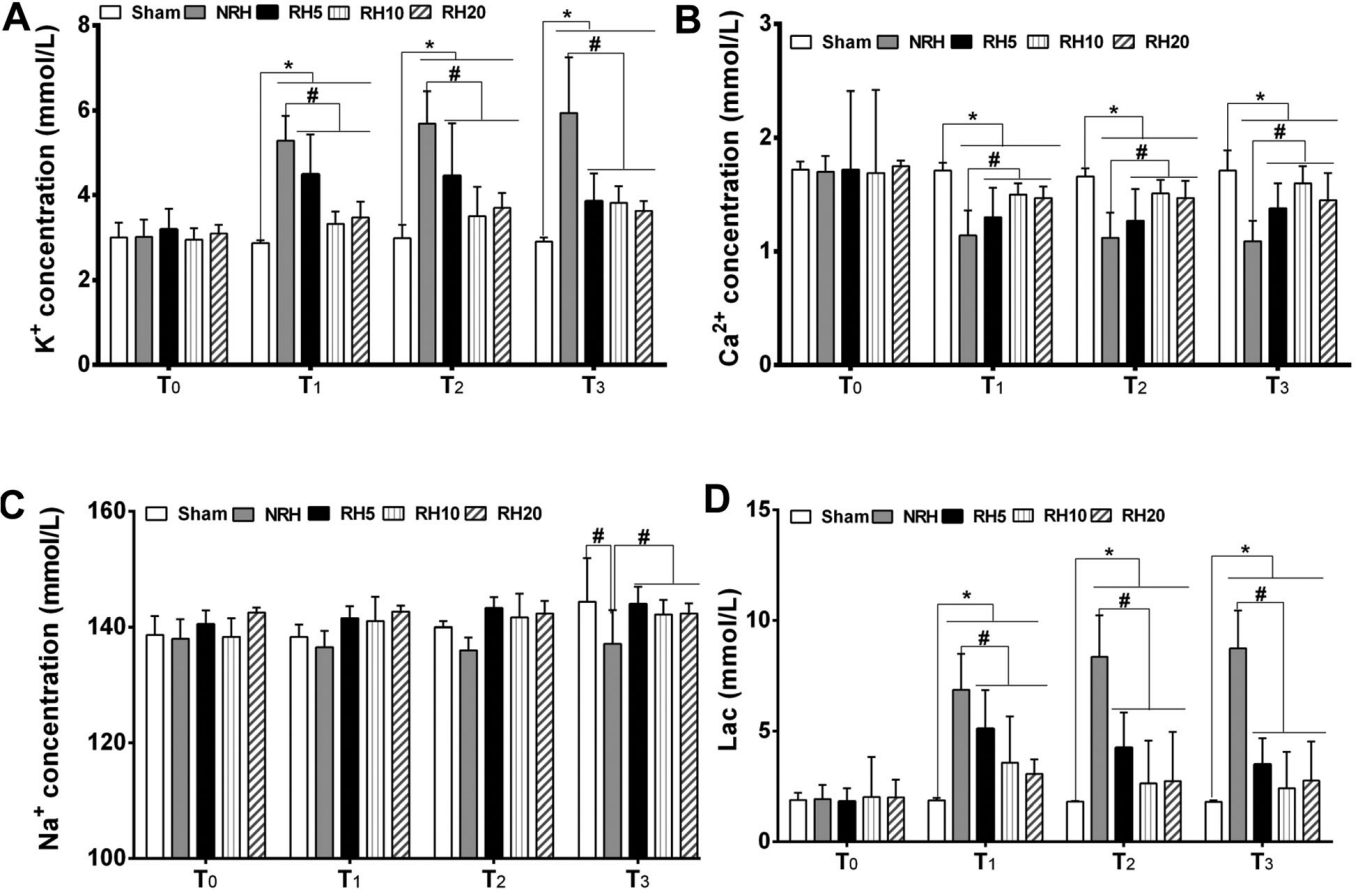
Blood gas analysis of lactic acid (Lac), and lectrolyte ion in serum. **a**. K^+^, **b**. Ca^2+^, **c**. Na^+^ and **d**. lactic acid (Lac) in arterial blood. **Notes:** Data are mean ± SD (*n* = 6). *p < 0.05 vs. Sham, #*p* < 0.05 vs. NRH

The electrolyte in arterial blood was also detected. Compared with sham group, the K^+^ concentration of NRH and RH groups was significantly elevated (*p < 0.05) after removing tourniquet, and then the concentration of K^+^ in RH groups was significantly lower than that of NRH group (#*p* < 0.05) (Fig. 4a). Similar effects were presented in detection of Ca^2+^ concentration. Compared with sham group, the Ca^2+^ concentration of NRH and RH groups was significantly decreased (*p < 0.05) after removing tourniquet, and then the concentration of Ca^2+^ in RH groups was significantly higher than that of NRH group (#*p* < 0.05) (Fig. 4b). The results of Na^+^ concentration displayed no statistical difference among all groups (Fig. 4c). Compared with sham group, the value of Lac in NRH and RH groups was significantly elevated (*p < 0.05) after removing tourniquet. However, the value of Lac in all RH groups was significantly lower than that of NRH group (#*p* < 0.05) (Fig. 4d). All those results demonstrated that RH could reduce the amount of lactic acid, mitigate the symptom of serum hyper-kalemia and hypo-calcemia induced by applying of tourniquet. Among the three RH groups, the effects of RH10 group were the most prominent.

### Change of ATPase activity, MDA in regional muscle and inflammatory cytokines in serum

**Fig. 5:**
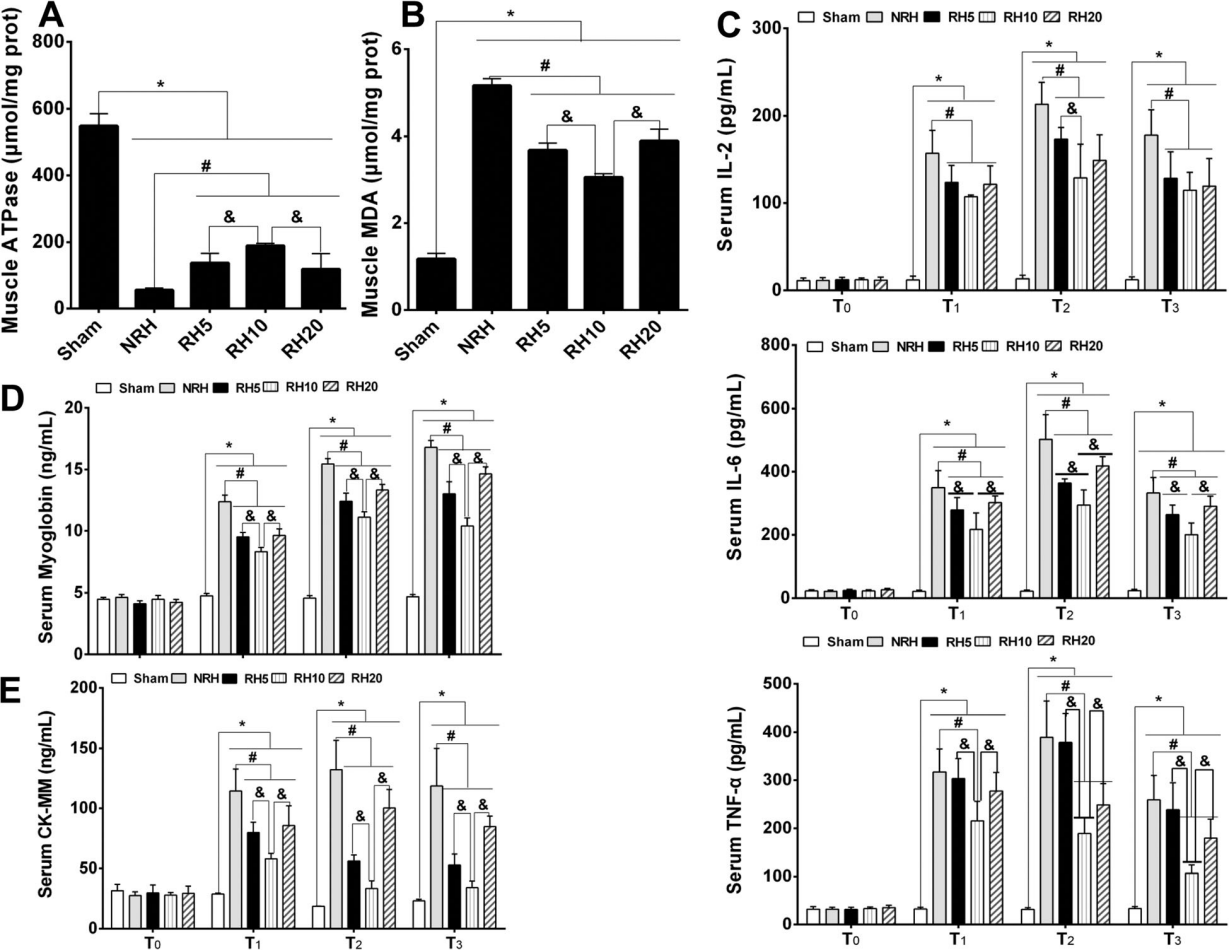
Change of ATPase activity, MDA in regional muscle and inflammatory cytokines in serum. **a**. ATPase activity of muscle. **b**. MDA content in muscle. **c**. Concentration of serum IL-2. **d**. Concentration of serum IL-6. **e**. Concentration of serum TNF-α. **f**. Concentration of serum myoglobin. **G**. Concentration of serum CK-MM. **Notes:** Data are mean ± SD (*n* = 6). *p < 0.05 vs. Sham, #*p* < 0.05 vs. NRH, &*p* < 0.05 vs. RH10

The ATPase activity in regional muscle significantly decreased (*p < 0.05) in NRH and RH groups compared with sham group. However, the RH group was significantly higher than NRH group (#p < 0.05), what’s more, RH10 group and the other two RH groups also had significant differences (&*p* < 0.05) (Fig. 5a). The change of MDA content in regional muscle showed opposite trend compared with that of ATPase activity. The MDA content significantly increased (*p < 0.05) in NRH and RH groups compared with sham group, among which the RH groups was lower than that of NRH group (#*p* < 0.05), especially, the RH10 group showed significant differences (&*p* < 0.05) compared with the other two RH groups (Fig. 5b).

The concentration of inflammatory cytokines (IL-2, IL-6, TNF-α) was detected in serum (Fig. 5c-e). The three inflammatory factors’ content showed no statistical significance among all groups before injuring (T_0_). After removing tourniquet (T_1_, T_2_, T_3_), all the three inflammatory cytokines significantly elevated except sham group, but the values in RH subgroups were found significantly lower than that of NRH group (#*p* < 0.05), the most prominent group was RH10 group.

### Change of myoglobin and CK-MM in serum

The concentration of myoglobin and CK-MM in serum was kept at normal ranges in all groups before injury (T_0_) (Fig. 5f and g). However, the content obviously increased (*p < 0.05) after removing tourniquet (T_1_, T_2_, T_3_) expect sham group. While the myoglobin and CK-MM content in RH groups were found significantly lower (#*p* < 0.05) than that of NRH group, and the RH10 group was the most prominent group. Among the three RH subgroups, the most prominent was RH10 group, which showed significant differences (&*p* < 0.05) compared with other two subgroups.

### The effect of Heme and HO-1 on distant lung injury

**Fig. 6:**
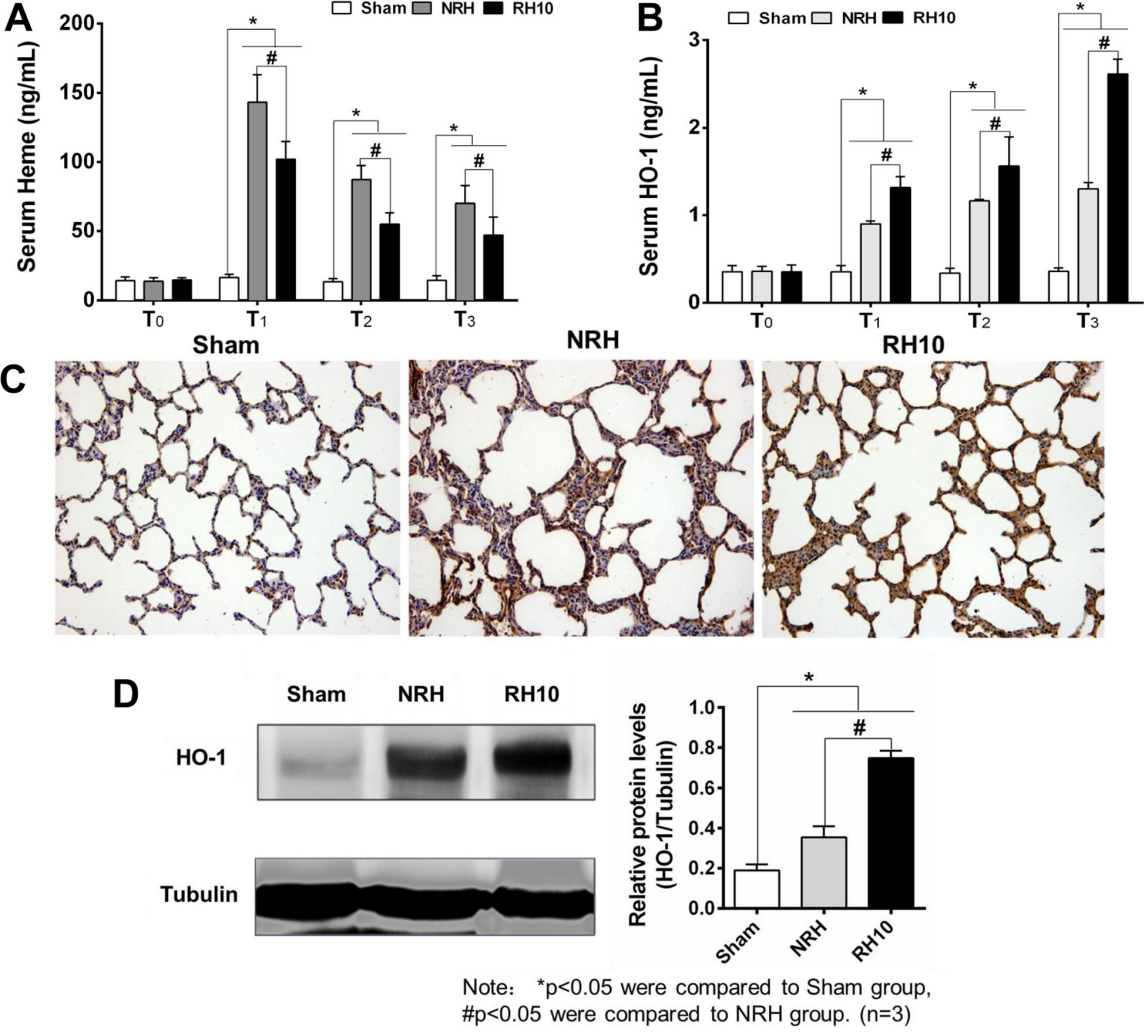
The effect of Heme and HO-1 on distant lung injury. **a**. Content of serum Heme. **b**. Content of serum Heme oxygenase 1 (HO-1). **c**. Immunohistochemistry analyses of HO-1 in lung. Representative microphotographs (20×) were taken from the Sham, NRH and RH10°C, respectively. **d**. Western bolt analyses of HO-1 in lung. **Notes:** Data are mean ± SD (*n* = 3). *p < 0.05 vs. Sham, #*p* < 0.05 vs. RH10

The HO-1 was a catalyst in the degradation of Heme [27], and Heme was one of the important mediators for inducing lung injury [28]. In this study, both the HO-1 and Heme were detected in serum, moreover, HO-1 in lungs was detected by western-bolt and immunohistochemistry. The content of HO-1 and Heme in all groups maintained at the same level before injury (T_0_), but increased significantly in the NRH and RH10 groups after removing tourniquet (T_1_, T_2_, T_3_). Noteworthy, the Heme value in RH10 group was significantly lower (#*p* < 0.05) than that of NRH group, and the HO-1 content in RH10 group was significantly higher (#*p* < 0.05) than that of NRH group (Fig. 6a and b). HO-1 could reduce total Heme content in injured rabbits, and also make protection effects for lung of injured rabbits.

Immunohistochemistry of HO-1 in lung showed that obviously positive signal was found in NRH and RH10 groups, especially in RH10 group, however weak signal was found in sham group (Fig. 6c). Thicker alveolar septum was presented both in NRH and RH10 groups compared with sham group, especially in NRH group. Similar results were verified in western-blot of HO-1. Compared with sham group, the expression of HO-1 in NRH and RH10 groups was detected significantly elevated (*p < 0.05), and HO-1 expression in RH10 group was significantly higher than that of NRH group (#*p* < 0.05) (Fig. 6d). All these results revealed that RH could obviously attenuate oxidative stress induced by applying of tourniquet.

## Discussion

Limbs injuries are extremely common in war-wounds or civil injuries. Tourniquet is considered as the first choice for controlling bleeding in limb injury, however, as mentioned above, the tourniquet application is limited by application-duration and application-occasion. Therefore, in this study, in order to get a more realistic trauma simulation to fragment injury combining with hemorrhage shock in battlefield or civilian life, rabbits were exsanguinated from femoral artery after injuring the soft tissue, due to the femoral artery has good elasticity and not easy to fracture, and furthermore, the hemorrhage in femoral artery is difficult to control once hitting.

### RH had a “long-term mitigation effects” on time-out application of tourniquet

According to the reports, limb injury combined with hemorrhage shock is a leading cause of preventable death on the battlefield, timely and correct use of tourniquet is a simple method to eliminate preventable death [29]. However, inappropriate (time-out use or use after hemorrhagic shock, or other improper operation) application of tourniquets on casualty could lead to organ injury, amputation, and even death [6].

Although hypothermia has been proposed in clinical treatment [14], it is rarely reported in treatment of war wounds or emergency surgery, controversial opinions are still held because of “lethal triad” theory, which demonstrate that hypothermia can aggravate acidosis and coagulopathy, then cause death of the patient [30, 31]. Previous study indicated that RH could attenuate pulmonary injury after hepatic ischemia and reperfusion [18, 19]. Furthermore, transient RH (15–20 °C) had been applied in trauma rats’ muscle treatment, which were subjected to blast injury, and protective effects were shown both on local traumatic tissue and distant organ injury [10, 11]. According to the literature, we speculated whether analogous protective effects of RH were also effective in limb fragment-penetrating trauma combined hemorrhagic shock for timeout-application of tourniquet.

In this study, three different gradients (5 ± 1 °C, 10 ± 1 °C and 20 ± 1 °C) of RH were employed on fragment injured rabbit limb which was employed tourniquet for 4 h, in the state of hemorrhagic shock. Compared with NRH group, mitigation effects of injury were demonstrated in all three RH groups. The injury of local muscles and distant-lungs were attenuated in RH groups, histological structure presented less hemorrhage area in RH groups compare with NRH, and the RH10 group was the most effective (Fig. 3a). The K^+^ and Ca^2+^ are the highest content of intracellular fluid cationic. When body is experienced traumatic and hemorrhagic shock, the increase of K^+^ content and the decrease of Ca^2+^ content in serum could cause cardiac arrest and failure of cardiopulmonary resuscitation, which have potentially life-threatening nature [32]. In this study, the significant decrease of K^+^ content and increase of Ca^2+^ content in serum were shown in RH groups compared with that of NRH group (Fig. 4a and b). Studies have shown that high content of inflammatory cytokines, CM- MM and myoglobin in serum could lead to lung injury directly or indirectly [33–35]. Myoglobin precipitation or accumulation have been proposed to cause oxidant injury and even to cause acute renal failure (ARF) after crush injuries/reperfusion [36, 37]. In this study, the levels of inflammatory cytokines (TNF-α, IL-6, IL-2), myoglobin and CK-MM in serum all decreased in RH groups compared with that of NRH group, and the most significant decline group was RH10 group (Fig. 5c-g).

It was shown that different level of protective effects was shown both on local traumatic muscle and distant-lung injury in RH group after removing tourniquet for 6 h. All of these indicated that transient RH (4 h) had a “long-term mitigation effects” (6 h) on time-out application of tourniquet.

### RH could improve energy-metabolism related protective effect and inhibit oxidative stress

The oxidative stress reaction in injured muscles was mitigated, which was demonstrated with increase of ATPase activity and decrease of MDA content in RH groups compared with NRH group, and the RH10 group was the most significant among three RH groups (Fig. 5a and b). The results above indicated that appropriate RH could regulate metabolism of ATP, inhibit cell and ATP metabolism, reduce the content of MDA and inflammatory factors, therefore improving energy-metabolism and playing oxidation protective effect.

Furthermore, previous reports have shown that myoglobin precipitation [36, 37] and excess Heme in tissue are important factors causing oxidative stress and inflammatory injury [38–40]. HO-1 is the rate-limiting enzyme in the oxidative breakdown of Heme, and catalyzes the conversion of Heme to carbon monoxide (CO), ferrous iron and biliverdin, which all have strong antioxidant effect. Strong and rapid expression of HO-1 induced by oxidative stress (such as Heme, interleukins, hydrogen peroxide) has been reported in the lung, liver, and kidney [41]. Besides, the expression of HO-1 could also be induced by low temperature and ischemia reperfusion, and protect the injured stimulation of tissues and organs [42, 43]. The results in this study indicated that lower levels of Heme were found in RH10 group compared with NRH group, and higher levels of HO-1 in lung were displayed in RH10 group than that of NRH group (Fig. 6). Besides, the decrease of serum myoglobin also was shown in RH groups compared with that of NRH group, and the RH10 group had the largest decline among the three RH groups (Fig. 5f). So the injury of distant-lung was relieved (Fig. 3a). All these results were in accordance with the previous reports mentioned above.

Consequently, RH could up-regulate the expression of HO-1 and catalysis decomposition of excess Heme or myoglobin, thereby inhibiting oxidative stress. Moreover, results in this study demonstrated that HO-1 played a critical role in “long-term mitigation effects” of RH, the probable reason is that HO-1 can improve oxidative stress reaction by regulating the metabolism of myoglobin and Heme, but the specific mechanism is still unknown.

## Conclusions

In summary, we could conclude that with the fluid resuscitation and core temperature maintenance, transient RH (4 h) had a “long-term mitigation effects” (continued for 6 h) on time-out application of tourniquet. Histopathological changes of lung and local muscle were significantly attenuated in RH groups compared with that in NRH group. Protective effects of regional limb hypothermia on local muscle and distant lung injury were indicated. These results showed that RH could mitigate the symptom of serum hyper-kalemia and hypo-calcemia, decrease skeletal muscle metabolism and myoglobin release, inhibit inflammatory responses and oxidative stress caused by prolonged application of tourniquet, finally reduce the injury of local muscle and distant lung. Particularly, among the three different RH, the most effective regional limb hypothermia was probably 10 °C. In conclusion, RH could attenuate secondary-injury caused by time-out application of tourniquets following limb fragments injury combined with hemorrhagic shock.

## Supplementary information

**Additional file 1: Table S1.** Physiological Variables During the Course of Study. (mean ± SD, *n* = 6)

## Data Availability

The datasets used and/or analysed during the current study are available from the corresponding author on reasonable request.

## References

[1] Stanec Z, Skrbic S, Depina I, Hulina D, Ivrlac R, Unusic J, Montani D, Prpic I, Unusic I (1993). Surgical management of war injuries involving soft tissue defects. Lijec Vjesn.

[2] Kelly JF, Ritenour AE, McLaughlin DF, Bagg KA, Apodaca AN, Mallak CT, Pearse L, Lawnick MM, Champion HR, Wade CE (2008). Injury severity and causes of death from Operation Iraqi Freedom and Operation Enduring Freedom: 2003–2004 versus 2006. J Trauma.

[3] Oragui E, Parsons A, White T, Longo UG, Khan WS (2011). Tourniquet use in upper limb surgery. Hand..

[4] Sadri A, Braithwaite IJ, Abdul-Jabar HB, Sarraf KM (2010). Understanding of intra-operative tourniquets amongst orthopaedic surgeons and theatre staff--a questionnaire study. Ann R Coll Surg Engl.

[5] Fitzgibbons PG, DiGiovanni C, Hares S, Akelman E (2012). Safe tourniquet use: a review of the evidence. J Am Acad Orthop Sur.

[6] Kragh JF, Walters TJ, Baer DG, Fox CJ, Wade CE, Salinas J, Holcomb JB (2009). Survival with emergency tourniquet use to stop bleeding in major limb trauma. Ann Surg.

[7] Noordin S, McEwen JA, Kragh JF, Eisen A, Masri BA (2009). Surgical tourniquets in orthopaedics. J Bone Joint Surg Am.

[8] Murphy CG, Winter DC, Bouchier-Hayes DJ (2005). Tourniquet injuries: pathogenesis and modalities for attenuation. Acta Orthop Belg.

[9] Feldman V, Biadsi A, Slavin O, Kish B, Tauber I, Nyska M, Brin YS (2015). Pulmonary embolism after application of a sterile elastic exsanguination tourniquet. Orthopedics..

[10] Zhao H, Ning J, Duan J, Gu J, Yi B, Lu K, Mo L, Lai X, Hennah L, Ma D (2014). Regional traumatic limb hypothermia attenuates distant hepatic and renal injury following blast limb trauma in rats. J Trauma Acute Care Surg.

[11] Ning J, Mo L, Zhao H, Lu K, Wang L, Lai X, Yang B, Zhao H, Sanders RD, Ma D (2014). Transient regional hypothermia applied to a traumatic limb attenuates distant lung injury following blast limb trauma. Crit Care Med.

[12] Menon M, Sood A, Bhandari M, Kher V, Ghosh P, Abaza R, Jeong W, Ghani KR, Kumar RK, Modi P (2014). Robotic kidney transplantation with regional hypothermia: a step-by-step description of the Vattikuti urology institute-Medanta technique (IDEAL phase 2a). Eur Urol.

[13] Sood A, McCulloch P, Dahm P, Ahlawat R, Jeong WJ, Bhandari M, Menon M (2016). Ontogeny of a surgical technique: robotic kidney transplantation with regional hypothermia. Int J Surg.

[14] Fan J, Cai S, Zhong H, Cao L, Hui K, Xu M, Duan M, Xu J (2017). Therapeutic hypothermia attenuates global cerebral reperfusion-induced mitochondrial damage by suppressing dynamin-related protein 1 activation and mitochondria-mediated apoptosis in a cardiac arrest rat model. Neurosci Lett.

[15] Nolan JP, Soar J, Zideman DA, Biarent D, Bossaert LL, Deakin C, Koster RW, Wyllie J, Bottiger B, Grp EGW (2010). European resuscitation council guidelines for resuscitation 2010 section 1 executive summary. Resuscitation..

[16] Santora RJ, Lie ML, Grigoryev DN, Nasir O, Moore FA, Hassoun HT (2010). Therapeutic distant organ effects of regional hypothermia during mesenteric ischemia-reperfusion injury. J Vasc Surg.

[17] Kragh JF, Baer DG, Walters TJ (2007). Extended (16-hour) tourniquet application after combat wounds: a case report and review of the current literature. J Orthop Trauma.

[18] Fu Z, Liu X, Geng B, Fang L, Tang C (2008). Hydrogen sulfide protects rat lung from ischemia-reperfusion injury. Life Sci.

[19] Patel S, Pachter HL, Yee H, Schwartz JD, Marcus SG, Shamamian P (2000). Topical hepatic hypothermia attenuates pulmonary injury after hepatic ischemia and reperfusion. J Am Coll Surg.

[20] Bozkurt NB, Moralioglu S, Vural IM, Sarioglu Y, Pekiner C (2008). Does tourniquet application alter the nitrergic responses of rabbit corpus cavernosum penis? A functional study. World J Urol.

[21] Chalidis BE, Kalivas E, Parziali M, Christodoulou AG, Dimitriou CG (2012). Cuff width increases the serum biochemical markers of tourniquet-induced skeletal muscle ischemia in rabbits. Orthopedics..

[22] Morykwas MJ, Howell H, Bleyer AJ, Molnar JA, Argenta LC (2002). The effect of externally applied subatmospheric pressure on serum myoglobin levels after a prolonged crush/ischemia injury. J Trauma.

[23] Jiang S, He X, Wang J, Zhou G, Zhang M, Ba L, Yang J, Zhao X (2013). Therapeutic mild hypothermia improves early outcomes in rabbits subjected to traumatic uncontrolled hemorrhagic shock. J Surg Res.

[24] Oncul S, Karabiyik L, Coskun E, Kadioglu E, Gulbahar O (2017). Comparisons of the effects of the sevoflurane and propofol on acute ischemia reperfusion and DNA damages in rabbits. Braz J Anesthesiol.

[25] Ning J, Mo L, Lu K, Lai X, Wang Z, Ma D (2012). Lung injury following lower extremity blast trauma in rats. J Trauma Acute Care Surg.

[26] Gu J, Chen J, Xia P, Tao G, Zhao H, Ma D (2011). Dexmedetomidine attenuates remote lung injury induced by renal ischemia-reperfusion in mice. Acta Anaesthesiol Scand.

[27] Tenhunen R, Marver HS, Schmid R (1969). Microsomal heme oxygenase. Characterization of the enzyme. J Biol Chem.

[28] Maguina P, Jean-Pierre F, Grevious MA, Malk AS (2008). Posterior interosseous branch palsy following pneumatic tourniquet application for hand surgery. Plast Reconstr Surg.

[29] Shlaifer A, Yitzhak A, Baruch EN, Shina A, Satanovsky A, Shovali A, Almog O, Glassberg E (2017). Point of injury tourniquet application during operation protective edge-what do we learn?. J Trauma Acute Care Surg.

[30] Mitra B, Tullio F, Cameron PA, Fitzgerald M (2012). Trauma patients with the 'triad of death'. Emerg Med J.

[31] Vardon F, Mrozek S, Geeraerts T, Fourcade O (2016). Accidental hypothermia in severe trauma. Anaesth Crit Care Pa.

[32] Epstein M, Ketteler M. Clinical and electrophysiological consequences of hyperkalemia. Nephrol News Issues. 2016. 27254900

[33] Elsayed N, Gorbunov N (2007). Pulmonary biochemical and histological alterations after repeated low-level blast overpressure exposures. Toxicol Sci.

[34] Horst K, Eschbach D, Pfeifer R, Relja B, Sassen M, Steinfeldt T, Wulf H, Vogt N, Frink M, Ruchholtz S (2016). Long-term effects of induced hypothermia on local and systemic inflammation-results from a porcine long-term trauma model. PLoS One.

[35] Plotnikov EY (2009). Myoglobin causes oxidative stress, increase of NO production and dysfunction of kidney's mitochondria. Biochimica Et Biophysica Acta Molecular Basis of Disease.

[36] Holt S, Moore K (2000). Pathogenesis of renal failure in Rhabdomyolysis: the role of myoglobin. Nephron Exp Nephrol.

[37] Murata I, Miyake Y, Takahashi N, Suzuki R, Fujiwara T, Sato Y, Inoe Y, Kobayashi J, Kanamoto I (2016). Low-dose sodium nitrite fluid resuscitation prevents lethality from crush syndrome by improving nitric oxide consumption and preventing myoglobin cytotoxicity in kidney in a rat model. Shock..

[38] Ryter SW, Tyrrell RM (2000). The heme synthesis and degradation pathways: role in oxidant sensitivity. Heme oxygenase has both pro- and antioxidant properties. Free Radic Biol Med.

[39] Wagener FA, Eggert A, Boerman OC, Oyen WJ, Verhofstad A, Abraham NG, Adema G, Van KY, De WT, Figdor CG (2001). Heme is a potent inducer of inflammation in mice and is counteracted by heme oxygenase. Blood..

[40] Mittal P, Shenoy S, Sandhu JS (2008). Effect of different cuff widths on the motor nerve conduction of the median nerve: an experimental study. J Orthop Surg Res.

[41] Constantin M, Choi AJS, Cloonan SM, Ryter SW (2012). Therapeutic potential of Heme Oxygenase-1/carbon monoxide in lung disease. Int J Hypertens.

[42] Peng TC, Jan WC, Tsai PS, Huang CJ (2011). Heme Oxygenase-1 mediates the protective effects of ischemic preconditioning on mitigating lung injury induced by lower limb ischemia-reperfusion in rats. J Surg Res.

[43] Zhao H, Mitchell S, Koumpa S, Cui YT, Lian Q, Hagberg H, Johnson MR, Takata M, Ma D (2016). Heme Oxygenase-1 mediates Neuroprotection conferred by argon in combination with hypothermia in neonatal hypoxia-ischemia brain injury. Anesthesiology..

