# Supplementary material for: Regional hypothermia attenuates secondary-injury caused by time-out application of tourniquets following limb fragments injury combined with hemorrhagic shock
Source: Scand J Trauma Resusc Emerg Med. 2019 Nov 21;27:104. doi: 10.1186/s13049-019-0678-3 (PMC6873525; doi:10.1186/s13049-019-0678-3)
Supplement: Supplementary file 1 — Additional file 1: Table S1. Physiological Variables During the Course of Study. (mean ± SD, n = 6) [file 13049_2019_678_MOESM1_ESM.docx]

Additional file 1

**Table S1.** Physiological Variables During the Course of Study. (mean ± SD, n=6)

|  | Group | | T_0_ | T_1_ | T_2_ | T_3_ |  |
| --- | --- | --- | --- | --- | --- | --- | --- |
| pH | | Sham | 7.40 ± 0.037 | 7.41 ± 0.05 | 7.34 ± 0.04 | 7.41 ± 0.02 | |
|  | | NRH | 7.43 ± 0.04 | 7.39 ± 0.06 | 7.38 ± 0.08 | 7.36 ± 0.07 | |
|  | | RH5°C | 7.45 ± 0.05 | 7.42 ± 0.06 | 7.41 ± 0.05 | 7.38 ± 0.06 | |
|  | | RH10°C | 7.41 ± 0.03 | 7.43 ± 0.40 | 7.41 ± 0.05 | 7.40 ± 0.04 | |
|  | | RH20°C | 7.44 ± 0.05 | 7.45 ± 0.03 | 7.40 ± 0.05 | 7.38 ± 0.06 | |
| PaCO_2_ (mmHg) | | Sham | 35.70 ± 6.59 | 29.27 ± 2.24 | 30.60 ± 2.34 | 27.60 ± 6.44 | |
|  | | NRH | 32.18 ± 3.80 | 26.65 ± 2.80 | 24.21 ± 2.08 | 22.26 ± 3.14 | |
|  | | RH5°C | 37.00 ± 4.92 | 24.47 ± 3.19 | 25.20 ± 1.99 | 26.25 ± 3.66 | |
|  | | RH10°C | 36.50 ± 3.08 | 24.79 ± 2.54 | 26.13 ± 2.23 | 25.97 ± 3.32 | |
|  | | RH20°C | 37.17 ± 2.40 | 28.16 ± 5.12 | 27.61 ± 4.27 | 27.47 ± 2.75 | |
| HCO_3_^-^ (mol/L) | | Sham | 22.13 ± 2.76 | 18.67 ± 0.93 | 17.40 ± 0.50 | 14.03 ± 4.33 | |
|  | | NRH | 21.68 ± 1.73 | 16.36 ± 3.08 | 14.65 ± 2.78 | 13.03 ± 3.17 | |
|  | | RH5°C | 25.60 ± 3.07 | 15.85 ± 2.06 | 15.73 ± 1.97 | 15.67 ± 0.88 | |
|  | | RH10°C | 22.94 ± 0.93 | 15.49 ± 2.28 | 15.87 ± 1.90 | 15.91 ± 2.25 | |
|  | | RH20°C | 25.20 ± 1.26 | 18.68 ± 4.41 | 17.05 ± 3.36 | 37.75 ± 5.67 | |
| SaO_2_% | | Sham | 96.33 ± 0.58 | 96.67 ± 0.58 | 96.00 ± 1.00 | 96.00 ± 1.00 | |
|  | | NRH | 95.75 ± 0.71 | 96.38 ± 1.85 | 96.63 ± 0.52 | 96.75 ± 0.46 | |
|  | | RH5°C | 96.00 ± 4.43 | 97.33 ± 1.21 | 96.83 ± 0.41 | 96.33 ± 0.52 | |
|  | | RH10°C | 95.57 ± 0.54 | 97.29 ± 0.49 | 96.86 ± 0.69 | 96.57 ± 0.54 | |
|  |  | RH20°C | 95.67 ± 3.14 | 97.17 ± 0.75 | 96.17 ± 1.60 | 96.00 ± 0.89 | |
| Breath rate (time/mim) | | Sham | 42.00 ± 2.65 | 45.00 ± 3.00^#^ | 45.00 ± 3.61^#^ | 47.67 ± 3.51^#^ | |
|  | | NRH | 42.33 ± 4.08 | 69.17 ± 19.00 | 67.67 ± 14.91 | 61.17 ± 10.15 | |
|  | | RH5°C | 46.17 ± 12.84 | 45.67 ± 10.44^#^ | 46.33 ± 15.11^#^ | 43.33 ± 13.35^#^ | |
|  | | RH10°C | 43.00 ± 4.52 | 42.50 ± 11.17^#^ | 47.83 ± 17.08^#^ | 42.83 ± 8.13^#^ | |
|  | | RH20°C | 44.00 ± 1.55 | 44.33 ± 3.27 | 44.33 ± 4.08 | 43.83 ± 2.32 | |
| Heart rate (time/mim) | | Sham | 258.50 ± 37.48 | 248.00 ± 22.63 | 253.00 ± 32.53 | 244.00 ± 32.53 | |
|  | | NRH | 257.50 ± 7.78 | 249.00 ± 16.64 | 252.50 ± 27.58 | 272.00 ± 12.73 | |
|  | | RH5°C | 237.67 ± 42.50 | 237.33 ± 37.10 | 256.83 ± 33.64 | 262.33 ± 18.07 | |
|  | | RH10°C | 253.33 ± 13.95 | 265.67 ± 21.78 | 257.33 ± 29.23 | 254.83 ± 17.31 | |
|  | | RH20°C | 264.67 ± 26.47 | 244.00 ± 25.81 | 243.67 ± 35.77 | 247.33 ± 41.57 | |
| Anal temperature (°C) | | Sham | 38.50 ± 0.35 | 38.70 ± 0.30 | 38.70 ± 0.70 | 38.33 ± 0.42 | |
|  | | NRH | 38.23 ± 0.56 | 38.68 ± 0.19 | 38.92 ± 0.42 | 38.77 ± 0.15 | |
|  | | RH5°C | 38.28 ± 0.62 | 38.70 ± 0.46 | 38.67 ± 0.41 | 38.52 ± 0.18 | |
|  | | RH10°C | 38.18 ± 0.23 | 38.68 ± 0.27 | 38.53 ± 0.31 | 38.62 ± 0.22 | |
|  | | RH20°C | 38.00 ± 0.50 | 38.20 ± 0.35 | 38.45 ± 0.37 | 38.50 ± 0.40 | |
| MAP (mmHg) | | Sham | 97.67 ± 9.07 | 84.00 ± 5.20^#^ | 86.67 ± 7.51^#^ | 83.67 ± 8.50^#^ | |
|  | | NRH | 95.86 ± 3.98 | 67.67 ± 14.91 | 61.17 ± 10.15 | 53.00 ± 11.64 | |
|  | | RH5°C | 92.33 ± 7.76 | 75.00 ± 11.76^#^ | 72.17 ± 12.39^#^ | 72.17 ± 11.36^#^ | |
|  | | RH10°C | 92.17 ± 9.24 | 77.67 ± 3.27^#^ | 76.67 ± 65.57^#^ | 74.33 ± 4.93^#^ | |
|  | | RH20°C | 97.00 ± 8.15 | 88.33 ± 10.67^#^ | 86.67 ±11.60^#^ | 83.00 ± 12.05^#^ | |

#p<0.05 vs. NRH.
